# Textile Pattern-Reconfigurable Antennas for 2.45 GHz Off-Body and On-Body Communications

**DOI:** 10.3390/mi17101123

**Published:** 2026-09-27

**Authors:** Jie Cui, Jian-Peng Chen, Yu-Xuan Qi, Feng-Xue Liu, Le Zhang, Hong-Fei Liu, Lei Zhao

**Affiliations:** 1 School of Transportation Engineering, Jiangsu University of Architectural Technology, Xuzhou 221116, China; star6108cj@163.com; 2 School of Physics and Electronic Engineering, Jiangsu Normal University, Xuzhou 221116, China; 2020251567@jsnu.edu.cn (J.-P.C.); qiyuxuan@jsnu.edu.cn (Y.-X.Q.); zhangle@jsnu.edu.cn (L.Z.); 3 Jiangsu Xiyi High-Tech New Materials Co., Ltd., Xuzhou 221400, China; xamri2019@163.com; 4 School of Information and Control Engineering, China University of Mining and Technology, Xuzhou 221116, China; leizhao@cumt.edu.cn

**Keywords:** textile antenna, pattern reconfiguration, microstrip patch antenna, monopole antenna, snap button

## Abstract

Electrical radiation pattern reconfiguration for textile antennas using diode-based bias circuits is challenging, as most textile materials cannot withstand high soldering temperatures. Metallic snap buttons, widely used in garment manufacturing, can be readily integrated into textile antennas as mechanical pattern-reconfiguration switches. Accordingly, four sets of copper snap buttons are loaded on a square microstrip patch antenna (MPA). The antenna operates in fundamental patch and monopole modes under OFF and ON states, respectively, which yields distinct resonant frequencies for the two reconfigurable modes. Analysis of the simulated currents on the patch inspires two strategies to align both the patch-mode and monopole-mode resonant frequencies at 2.45 GHz. The first employs two parallel slots between the feeding probe and snap buttons to extend the monopole-mode current path while maintaining the patch-mode current path unchanged. The second increases the patch width to achieve similar current-path tuning. Two textile antennas are designed and fabricated following the two strategies. Simulations and prototype measurements validate the 2.45 GHz pattern reconfiguration of the two designs and further verify their robustness against human-body loading and bending deformation.

## 1. Introduction

As a research hotspot in antenna design, wearable antennas have been extensively investigated and deployed in diverse fields, including medical monitoring, athletic training assistance, and the defense industry [1,2,3,4,5,6,7,8]. Textile antennas are fabricated using textile materials, such as conductive fabrics and threads, and their light weight, high flexibility, and excellent compatibility with clothing well meet the physical requirements for wearable antennas [7,8,9,10,11]. On the other hand, off-body and on-body communications in wireless body-area networks (WBANs) require wearable antennas to exhibit distinct radiation directions [12,13,14]. Omnidirectional antennas can satisfy the radiation direction requirements for both off-body and on-body links, but their ineffective radiation in non-target directions inevitably results in low utilization efficiency of the total radiation power. Conventional directional antennas can deliver most of their radiation power toward a fixed target direction, but fail to achieve multi-directional signal coverage. A wearable antenna with an adjustable radiation pattern can achieve flexible multi-directional signal coverage for different communication tasks while avoiding ineffective radiation in non-target directions, and thus can be spatially reused for both types of WBAN communications [15]. According to existing literature, pattern diversity and pattern reconfiguration are the two primary solutions for achieving an adjustable radiation pattern in a single wearable antenna.

Pattern diversity antennas based on multiple radiators or multi-eigenmode metasurfaces can achieve distinct radiation patterns at a fixed frequency by feeding different ports in their multi-port feeding networks. Reference [16] introduced a diversity antenna with two layered patches and two feeding ports for WBAN applications. At 2.45 GHz, the TM_01_ and TM_11_ modes were respectively excited on its two layered patches by switching the feeding port, and omniazimuthal/broadside radiation patterns were correspondingly achieved for off-/on-body applications. Reference [17] presented a pattern diversity patch antenna consisting of a notch-loaded ring with a coplanar slot-loaded circular patch within its aperture and two separate ports to excite broadside and conical radiation at 3.5 GHz. In references [18,19], two wideband pattern diversity antennas based on metasurfaces were designed, and distinct characteristic modes were excited by feeding different ports to achieve broadside and conical patterns at a fixed frequency. The diversity design is effective in switching the radiation pattern; however, its multiple radiators or metasurface structures inevitably increase the thickness or 2D footprint of the antenna, and the multi-port design also results in a complex feeding network structure.

Pattern-reconfigurable antennas are typically designed by integrating switching circuits into their radiating or feeding structures. The ON/OFF states of these switching circuits are controlled via bias networks or mechanical switches to reconfigure the antenna’s operating mode and radiation pattern. In reference [20], two wearable antennas were proposed with diode-based bias circuits integrated into their feeding network and radiating patch, respectively, and the omnidirectional and directional radiation patterns were achieved at 2.45 GHz by applying distinct bias voltages. In reference [21], a flexible conformal pattern-reconfigurable antenna constructed from a PDMS-conductive fabric composite was presented. The states of four PIN diodes were switched by varying the bias voltages, enabling reconfiguration between broadside and monopole-like modes/patterns at 5.13 GHz. In reference [22], patch-like and monopole-like radiation patterns were separately realized at 2.45 GHz by switching the diode states within four switching circuits integrated on a wearable patch antenna. In reference [23], snap-button modules incorporating RF switching circuits were loaded onto a flexible textile patch antenna, and the antenna exhibited omnidirectional and broadside radiation patterns for on-body and off-body communications at 2.45 GHz, respectively. Furthermore, reference [24,25,26,27] reported several rigid antennas employing similar switching circuits to reconfigure their operating modes and radiation patterns at a fixed frequency. Compared with diversity-based design, the pattern-reconfiguration strategy does not necessarily require a multi-port feeding network. However, owing to the low thermal resistance of most conductive textiles at high temperatures, directly soldering circuit components such as PIN diodes onto fabric radiators or ground planes is challenging. Accordingly, the aforementioned wearable mode/pattern-reconfigurable antennas often adopt non-textile materials such as copper tape/foil for their radiating elements and ground planes, or utilize standalone button-integrated modules incorporating electronically controlled switching circuits. This limits the practical applications of electronically controlled mode/pattern-reconfigurable designs in textile antennas. In our previous work [28], metallic snap buttons, which are widely used in garment manufacturing, were loaded on a textile coupled-mode substrate-integrated cavity (CMSIC) antenna as mechanical switches to allow simultaneous shifts of its native closely-spaced even- and odd-mode resonances. The radiation patterns for each mode were almost unchanged during frequency tuning, and different patterns could be obtained at a fixed frequency by aligning both the even-mode resonance frequency and the shifted odd-mode resonance frequency at 2.45 GHz.

This article presents two textile pattern-reconfigurable antennas for 2.45 GHz off-body and on-body communications. A square microstrip patch antenna (MPA) with four pairs of metallic snap buttons is first designed. Its patch/monopole reconfiguration characteristic is investigated through HFSS simulations, and the distinct resonance frequencies for the patch and monopole modes are analyzed using equivalent circuits and theoretical deductions. Subsequently, a fixed-frequency pattern reconfiguration realization by adding slots is proposed based on the simulated patch currents and is validated through simulations and prototype measurements. Furthermore, an alternative strategy for 2.45 GHz pattern reconfiguration by increasing patch width is developed and verified by simulated and measured results. The snap-button switching configuration adopted in this article is inspired by our prior work [28], yet the underlying principle for radiation pattern reconfiguration is distinct. Each of the proposed antennas excites only a single resonance under each button state, and the resulting distinct current distributions produce different radiation patterns. Different from [28], the snap buttons here reconfigure the antenna geometries themselves, rather than tuning the resonant frequencies of two inherent modes.

## 2. Patch/Monopole Reconfiguration Based on Square MPA with Metallic Snap Buttons

A basic square MPA is first designed as shown in Figure 1a. PF-4 foam is selected as the substrate, considering its high flexibility and excellent low-loss performance (dielectric loss tangent tan *δ* = 0.0001). Its thickness *h* and relative permittivity *ε_r_* are 3.2 mm and 1.06, respectively. The bottom surface of the substrate is fully covered with copper/nickel-coated polyester fabric, which has a measured surface resistance of 0.04 Ω\sq, acting as the ground plane. The size of the ground plane is set to 100 × 100 mm^2^ to isolate the antenna radiation and human tissues. The square patch (*l_p_* = *w_p_*) is made of the same conductive fabric and attached to the top of the substrate with its center coinciding with that of the substrate. A copper cylinder with a radius of 0.6 mm acts as the feeding probe. A circular hole with a radius of 1.5 mm is cut from the ground plane to avoid direct contact between the probe and the ground.

Based on the classic transmission-line model [29], *l_p_* is calculated and optimized to 53.5 mm to achieve a fundamental-mode (TM_10_) resonance at 2.45 GHz, with the feeding position *x_f_* determined to be 9.5 mm via HFSS simulations for impedance matching. Figure 2 presents the simulated return loss (|S_11_|) curve of the basic square MPA.

Inspired by reference [22], it is assumed that adding four symmetrical shorting vias is expected to convert the square MPA into a monopole-like antenna. In this work, copper snap buttons are employed as reconfigurable shorting vias, owing to their good compatibility with textile antennas [28,30]. A square MPA integrated with snap buttons is thus constructed as shown in Figure 1b,c, and is designated as Ant I. As depicted in the insets of Figure 1, the male snap buttons are mechanically attached to the fabric ground plane, while four circular holes with a radius of 2.5 mm are etched in the patch. Accordingly, the patch is electrically isolated from the male buttons when the female buttons are detached (button-OFF state), whereas the patch is shorted to the male buttons when the female buttons are fastened (button-ON state). With the values of *l_p_* and *x_f_* kept unchanged, the position parameter of the snap buttons *x_b_* is optimized to 17.5 mm to ensure satisfactory impedance matching in both the button-OFF and button-ON configurations. The simulated |S_11_| responses of Ant I in the OFF and ON states are plotted in Figure 2, yielding resonant frequencies of 2.34 GHz and 2.63 GHz, respectively.

In the button-OFF state, Ant I operates as a conventional MPA, whose equivalent RLC circuit is established and presented in Figure 3a [29]. The input impedance *Z_in_* is entirely determined by the patch impedance $Z_{p}$. $Z_{p}$ and its imaginary part, i.e., the reactance $X_{p}$, can be expressed as follows:

(1) $$Z_{p}=\frac{R_{p}(Z_{C_{p}}//Z_{L_{p}})}{R_{p}+(Z_{C_{p}}//Z_{L_{p}})}=\frac{jL_{p}R_{p}^{2}+L_{p}R_{p}\frac{L_{p}}{C_{p}}\left(\frac{2\pi fC_{p}}{1-4\pi^{2}f^{2}L_{p}C_{p}}\right)}{\left[C_{p}R_{p}^{2}+\frac{L_{p}^{2}}{C_{p}}{\left(\frac{2\pi fC_{p}}{1-4\pi^{2}f^{2}L_{p}C_{p}}\right)}^{2}\right]\left(\frac{2\pi fC_{p}}{1-4\pi^{2}f^{2}L_{p}C_{p}}\right)}$$

(2) $$X_{p}=im(Z_{p})=\frac{L_{p}R_{p}^{2}C_{p}\left(\frac{2\pi fC_{p}}{1-4\pi^{2}f^{2}L_{p}C_{p}}\right)}{C_{p}^{2}R_{p}^{2}+{L_{p}^{2}\left(\frac{2\pi fC_{p}}{1-4\pi^{2}f^{2}L_{p}C_{p}}\right)}^{2}}$$

Based on (2), a graphical sketch of $X_{p}$ versus frequency is drawn in Figure 4. The curve exhibits an abrupt transition from inductive to capacitive infinity at $f_{0}=1/2\pi \sqrt{L_{p}C_{p}}$, which corresponds to the resonance frequency of the fundamental patch mode.

On the other hand, when the buttons are ON, *Z_in_* comprises the patch in series with the ON-state buttons, as shown in the equivalent circuit in Figure 3b [31]. Accordingly, the reactance associated with the button part $X_{b}$ can be expressed as:

(3) $$X_{b}=\frac{L_{b}R_{b}^{2}C_{b}\left(\frac{2\pi fC_{b}}{1-4\pi^{2}f^{2}L_{b}C_{b}}\right)}{C_{b}^{2}R_{b}^{2}+{L_{b}^{2}\left(\frac{2\pi fC_{b}}{1-4\pi^{2}f^{2}L_{b}C_{b}}\right)}^{2}}$$

Considering the significantly smaller size of the metallic buttons compared to the patch, the intrinsic resonance frequency of the button part *f*_1_ is obviously much higher than that of the patch part *f*_0._ Therefore, the curve of −$X_{b}$ is plotted in Figure 4 based on (3), showing a transition from capacitive to inductive infinity at *f*_1_. In this case, the total reactance of the Ant I must be zero to achieve impedance matching, and thus $X_{p}$ should equal −$X_{b}$. From DC (*f* = 0) to *f*_1_, −$X_{b}$ remains negative, and the frequency at which $X_{p}$ = −$X_{b}$ is thus higher than *f*_0_. This theoretically explains the higher simulated resonance frequency of Ant I with buttons ON compared to the OFF scenario.

Figure 5 shows the simulated |S_11_| curves of Ant I with snap buttons embedded at different positions. It can be observed that varying *x_b_* does not induce a significant shift in the resonance frequency or severe degradation in impedance matching with buttons OFF, but leads to considerable deterioration in impedance matching performance when buttons are ON. Therefore, parameters *l_p_* and *x_f_* should first be optimized in the button-OFF state to achieve impedance matching at the target resonance frequency, and parameter *x_b_* can be subsequently optimized for impedance matching in the button-ON configuration.

The vector current on the patch (J_surf_), internal electric field (E-field), and coplanar-polarized radiation patterns of Ant I are simulated and shown in Figure 6. When the snap buttons are OFF and ON, the simulations show typical results of the fundamental patch and monopole modes, respectively. The radiation pattern in the patch mode is suitable for off-body communications with a main beam along the +*z* direction, whereas that in the monopole mode is appropriate for on-body communications, featuring two main beams between the +*z* axis and the *xy* plane. However, the distinct resonance frequencies of the two modes make unsuccessful mode/pattern reconfiguration at a fixed frequency. Therefore, the key strategy to achieve fixed-frequency pattern reconfiguration for Ant I is to align the resonance frequencies of the two switchable modes.

## 3. Fixed-Frequency Pattern Reconfiguration Realization by Adding Slots

### 3.1. Geometry Design and Parameter Analysis

Based on the simulated current distributions in Figure 6, two dashed lines can be marked in the figure, and they are intersected by the button-OFF currents but are parallel to the button-ON currents. Inspired by our previous work [32], it is assumed that adding slots along these dashed lines lowers the resonance frequency of Ant I in the monopole mode without changing that in the patch mode. Accordingly, a slot-based method for fixed-frequency mode/pattern reconfiguration is proposed. As shown in Figure 7, two identical straight slots are symmetrically added on the patch, and the proposed configuration is denoted as Ant II. The substrate thickness *h* and ground plane dimensions remain unchanged from those given in Section 2.

Parametric analysis is carried out using simulations for the dimensions and positions of the slots as shown in Figure 8. For the button-OFF scenario, varying *l_s_*, *w_s_*, or *d* barely induces obvious shifts of the resonance frequency or degradation of the impedance matching, owing to the parallel alignment between the slots and the currents on the patch. For the button-ON scenario, however, varying these parameters results in degradation of the impedance matching. When *d* decreases from 6 mm to 4 mm, the resonance frequency of the monopole mode is effectively reduced. However, when *d* is further reduced, the female buttons partially cover the slots, and the effective slot length is reduced. As a result, the monopole-mode resonance frequency unexpectedly jumps upward, and the impedance matching is dramatically deteriorated. In order to align the resonance frequencies of Ant II in both modes at 2.45 GHz, the dimensional parameters are optimized, and their optimized values are listed in Table 1.

### 3.2. Simulation Analysis

The simulated |S_11_| curves of Ant II in free space are shown in Figure 9, and both resonance frequencies with buttons OFF and ON are 2.45 GHz. The simulated patch-mode and monopole-mode relative −10 dB impedance bandwidths are 2.8% and 12%, respectively. Figure 10 shows the simulated coplanar-polarized gain patterns of Ant II. The simulated maximum coplanar-polarized gain at 2.45 GHz is 9.0 dBi in the patch mode with buttons OFF and 4.4 dBi in the monopole mode with buttons ON, and the simulated radiation efficiencies at 2.45 GHz for both states are above 97%. The simulated results of Ant II illustrate its mode/pattern reconfiguration characteristics at 2.45 GHz.

In order to investigate the influence of the human body on the antenna performance, a 300 × 300 × 60 mm^3^ phantom is modeled to imitate the human tissues, including skin, fat, and muscle. The thicknesses and electrical/physical characteristics of these tissue layers are obtained from reference [33] and listed in Table 2. The simulated |S_11_| curves and 2.45 GHz coplanar-polarized gain patterns of Ant II are respectively shown in Figure 9 and Figure 10. In the patch mode with buttons OFF, the overlap between the on-phantom and free-space|S_11_| curves indicates sufficient electromagnetic isolation owing to the ground plane. In the monopole mode with buttons ON, however, the surface currents on the patch can flow into the ground plane through these metallic buttons, and the currents on the ground also contribute to the antenna radiation. Therefore, the current path and equivalent impedance of the antenna suffer more severe influences from the human tissues in the monopole mode than in the patch mode, where the ground does not contribute to the radiation. Such a radiation mechanism of the monopole mode explains the observed shifted resonance frequency (2.49 GHz) and deterioration of the impedance matching in the simulated on-phantom |S_11_| curve of Ant II. Furthermore, Ant II exhibits a much larger decrease in backward radiation on the phantom model than in free space due to the reflection effect of the phantom. Its radiation efficiency drops to 91% with the buttons OFF and 74% with the buttons ON, attributed to electromagnetic absorption of the phantom. The much larger decrease in the radiation efficiency of Ant II with buttons ON further illustrates the analysis of its radiation mechanisms in the two modes. Under human-phantom loading, the monopole-mode Ant II suffers a resonant-frequency shift and radiation efficiency reduction. Nevertheless, its −10 dB impedance band (2.37~2.58 GHz) still fully covers the 2.45 GHz ISM band (2.4~2.4835 GHz). Despite the efficiency drop to 74%, the residual radiation efficiency is still acceptable for low-power short-range on-body WBAN communication.

Figure 11 shows the simulated 1 g average SAR distributions of Ant II in the phantom model at 2.45 GHz with a standard input power of 0.5 W. The simulated maximum SAR is 0.12 W/kg with buttons OFF and 0.3 W/kg with buttons ON at 2.45 GHz, and well satisfies the safety requirements of IEEE C95.1-2005 (below 1.6 W/kg) [34] and EN 50361-2001 (below 2.0 W/kg) [35] in both scenarios.

### 3.3. Prototype Measurement Results

A textile prototype of Ant II as shown in Figure 12 is fabricated for experimental verification. An SMA connector is used to connect the antenna to the input cable, and the conductive epoxy CW2460 is employed to stick its base and probe to the fabric patch and ground plane. Ant II achieves the best level of patch size listed in Table 3 (0.19 $\lambda_{\varepsilon }^{2}$, where $\lambda_{\varepsilon }$ denotes the wavelength in the substrate), and offers the advantage of a single-layer patch compared to reference [16].

The |S_11_| curves of Ant II in free space are measured and drawn in Figure 9, with slight shifts from the simulated curves, which can be caused by minor fabrication errors. The measured free-space resonance frequencies with buttons OFF and ON align at 2.44 GHz, and the measured relative −10 dB impedance bandwidths are 2.7% and 12.0%, respectively. The |S_11_| curves of Ant II are also measured on a volunteer’s body, and written informed consent has been obtained from the volunteer prior to all on-body measurements. The cable connecting the SMA connector of the prototype to the vector network analyzer is tucked under the volunteer’s armpit, and the prototype is positioned above the chest and upper-arm region. The ground plane is separated from human skin by a cotton T-shirt; the antenna plane is kept approximately parallel to the body surface with a 5 mm average antenna-to-skin separation. The measured on-body|S_11_| curves of Ant II are also shown in Figure 9. The measured on-body button-OFF curve almost overlaps with the free-space curve, and the button-ON curve exhibits an increased resonance frequency (2.47 GHz) compared to the free-space scenario, as anticipated in the simulations. Additionally, the |S_11_| curves are measured when the flexible prototype is bent on a cylindrical surface with a radius of 10.5 mm in two orthogonal directions, as shown in the insets of Figure 12, and the measured results are also shown in Figure 9. When the buttons are OFF, the measured |S_11_| curves with bending conditions in both considered directions basically overlap with each other and exhibit the same resonance frequencies but lower notch depths compared to the flat scenario. When the buttons are ON, however, the measured resonance frequency under both bending conditions slightly increases to approximately 2.46 GHz due to the shortened effective current path, which is caused by the wrinkling of the ground plane.

The coplanar-polarized gain patterns of Ant II are measured in an anechoic chamber at 2.44 GHz. The measured patterns are presented in Figure 10 with the expected shapes for off-body communications (with buttons OFF) and on-body communications (with buttons ON). The measured maximum coplanar-polarized gain is 8.5 dBi with buttons OFF and 4.1 dBi with buttons ON. Due to the limited measurement equipment, the radiation efficiency and on-body patterns are not practically measured for the proposed antenna.

Owing to the adoption of the ultra-low-loss substrate and highly conductive fabric, Ant II achieves a leading level of radiation efficiency. Simulation models cannot fully account for practical losses such as contact-resistance loss and cable loss during measurements, and the simulated efficiency is generally higher than the measured counterpart. Therefore, directly comparing the simulated efficiency of Ant II and the measured efficiencies of some of the listed references on the same basis may overestimate the performance of the proposed antenna. Accordingly, the comparative conclusions in Table 3 are provided mainly for qualitative trend reference. To draw a rigorous quantitative conclusion, measured radiation efficiencies of the proposed antennas should be evaluated under identical test conditions in future work.

In terms of the maximum gain, the antenna structure presented in reference [25] incorporated driver and parasitic elements and was analogous to a Yagi–Uda antenna. In reference [27], the ground planes of the antennas did not participate in radiation in all operating modes. The proposed geometry of reference [28] operated as a two-element array. Ant II operates as a single-element radiator in both modes, and the fact that the currents on the ground plane contribute to back radiation in the monopole mode results in a relatively low forward radiation intensity. Consequently, the maximum gains of Ant II in certain modes are lower than those of the aforementioned studies, yet still outperform other reported designs in Table 3.

## 4. Fixed-Frequency Pattern Reconfiguration Realization by Increasing Patch Width

### 4.1. Geometry Design

According to the classic transmission-line model and simulated patch currents in Figure 6, increasing the patch width *w_p_* does not change the current path length of the fundamental patch mode, while that of the monopole mode increases obviously. Simulations of Ant I with *w_p_* = 63.5 mm are carried out for verification, and the simulated |S_11_| curves are shown in Figure 2. The button-ON resonance frequency is reduced to 2.35 GHz to overlap with the unchanged button-OFF resonance frequency, but the impedance matching exhibits a certain degradation for both OFF and ON scenarios. Figure 13 shows the simulated curves of the button-OFF and button-ON resonance frequencies with respect to *w_p_*. It is observed that increasing *w_p_* can effectively reduce the monopole-mode resonance frequency until it overlaps with the patch-mode counterpart, which essentially remains unchanged. Based on that, an alternative implementation method of fixed-frequency mode/pattern reconfiguration is proposed in this section by increasing the patch width of Ant I.

The improved antenna geometry is shown in Figure 14 and denoted as Ant III. The geometry has the same ground plane dimensions as Ant I and Ant II, and its patch length *l_p_* is re-optimized through simulations to resonate at 2.45 GHz with buttons OFF. Parameters *x_f_* and *x_b_* are optimized for impedance matching in the patch and monopole modes, respectively. Through simulations, parameter *w_p_* is also optimized to align the monopole-mode resonance frequency at 2.45 GHz. The optimized dimensional parameters are also listed in Table 1.

### 4.2. Simulation Analysis

Figure 15 shows the simulated |S_11_| curves of Ant III. The free-space simulation results show that Ant III has a resonance frequency of 2.45 GHz for both states of the buttons, and the relative −10 dB impedance bandwidth is 3.4% with buttons OFF and 7.9% with buttons ON. When Ant III is placed on the phantom model, the simulated |S_11_| curve remains essentially unchanged with buttons OFF, and the shift of the resonance frequency to 2.47 GHz and the degradation of impedance matching for the button-ON state can also be explained by the radiation mechanism of the monopole mode.

Figure 16 shows the simulated coplanar-polarized gain patterns of Ant III at 2.45 GHz. In free space, the simulated maximum coplanar-polarized gain is 9.2 dBi with buttons OFF and 4.2 dBi with buttons ON, and the simulated radiation efficiencies in both scenarios are above 99%. The simulated 2.45 GHz free-space patterns exhibit the expected shapes for off-body communications with buttons OFF and on-body communications with buttons ON. The on-phantom simulations of Ant III also show reduced backward radiation and decreased radiation efficiencies (93% in patch mode and 76% in monopole mode). Similarly, for Ant III under human-body loading, its −10 dB impedance band can still completely cover the 2.45 GHz ISM band, and its remaining efficiency satisfies the basic requirement of short-range low-power on-body WBAN communications.

Figure 17 shows the simulated SAR distributions of Ant III at 2.45 GHz with a 0.5 W input power. The results show that the SAR in the phantom is below 0.28 W/kg in both modes, and therefore illustrate the safety of Ant III for wearable use.

### 4.3. Prototype Measurements

A textile prototype of Ant III, as shown in Figure 18, is fabricated using the aforementioned materials and procedures. With a slightly larger patch size (0.22 $\lambda_{\varepsilon }^{2}$) compared with Ant II, Ant III is of an even simpler patch geometry without slots.

In free space, the measured resonant frequency of Ant III is 2.46 and 2.47 GHz with buttons OFF and ON, respectively. The measured −10 dB impedance bandwidth reaches 3.2% in the button-OFF state and 8.4% in the button-ON state. The measured maximum coplanar-polarized gain is 8.8 dBi with the button OFF and 3.8 dBi with the button ON, as listed in Table 3. Minor discrepancies between simulated and measured results can be attributed to fabrication tolerances of conductive fabric, SMA connector assembly errors, prototype manufacturing imperfections, and slight assembly inconsistency of metallic snap-button switches.

Measurements of the |S_11_| curves on the human body and on the cylindrical surface are also carried out for Ant III. The agreement between these measured curves and the unbent free-space results, as shown in Figure 15, further verifies the robust performance of Ant III against human body loading and physical deformations.

## 5. Conclusions

Two textile pattern-reconfigurable antennas have been proposed in this article. For both geometries, metallic snap buttons are employed as switches to reconfigure the operation mode and corresponding radiation pattern. Simulation results show that the basic square MPA with buttons operates in the patch/monopole mode with off-/on-body radiation patterns when the buttons are OFF/ON, respectively, but the corresponding resonance frequencies are also different. To align two resonance frequencies for the fixed-frequency reconfiguration, two strategies have been proposed and verified by simulations and prototype measurements. The first employs two slots on the patch that are parallel to the patch-mode currents but intersect with the monopole-mode currents, and the resonance frequency of the monopole mode is lowered to coincide with the unchanged resonance frequency of the patch mode. In the second solution, the patch width is increased to achieve similar impacts on the resonance frequencies of both modes. Two geometries are designed, fabricated, and measured to validate both strategies. According to simulated and measured results, the designed antennas exhibit advantageous patch sizes, radiation efficiencies, and maximum gains compared with other candidates. It should be noted that the mechanical snap button switch has inherent practical limitations. Repeated fastening and unfastening operations may introduce variations in contact resistance, which could shift antenna resonant frequency and deteriorate impedance matching. Future work will focus on improving the contact stability of snap buttons and carrying out cyclic reliability experiments.

## Figures and Tables

**Figure 1:**
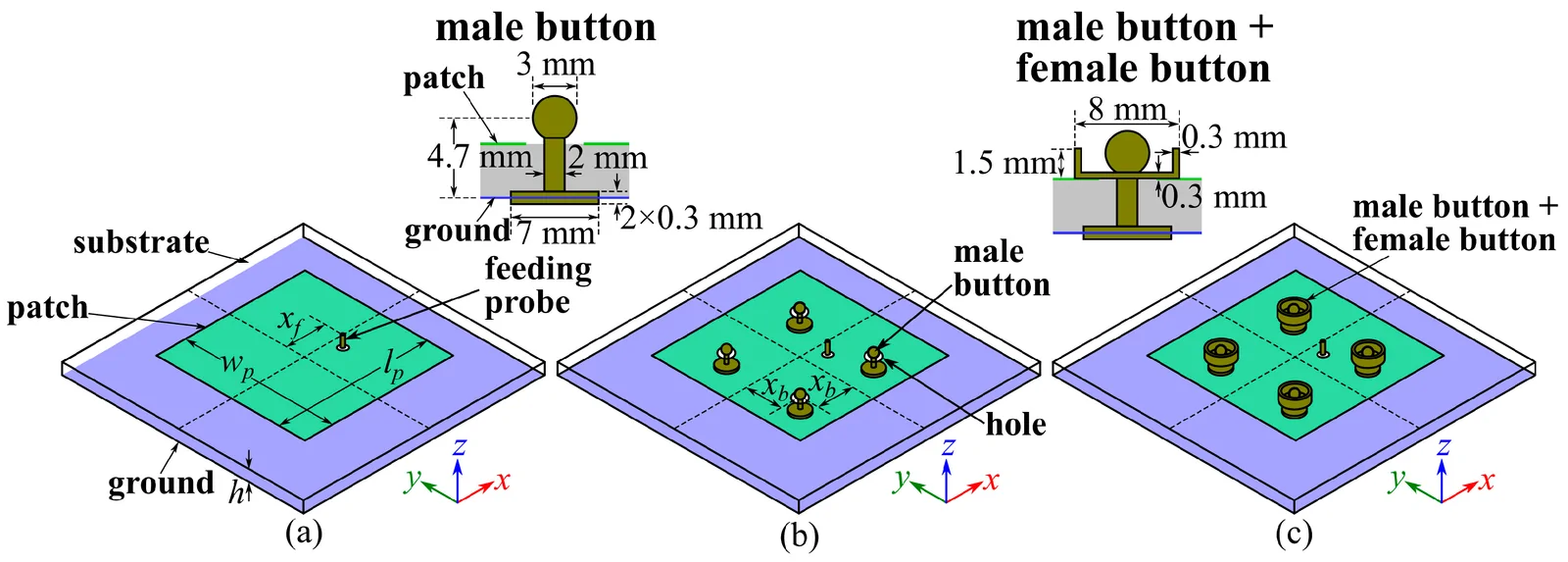
Geometries of (**a**) basic square MPA, (**b**) Ant I with buttons OFF, and (**c**) Ant I with buttons ON.

**Figure 2:**
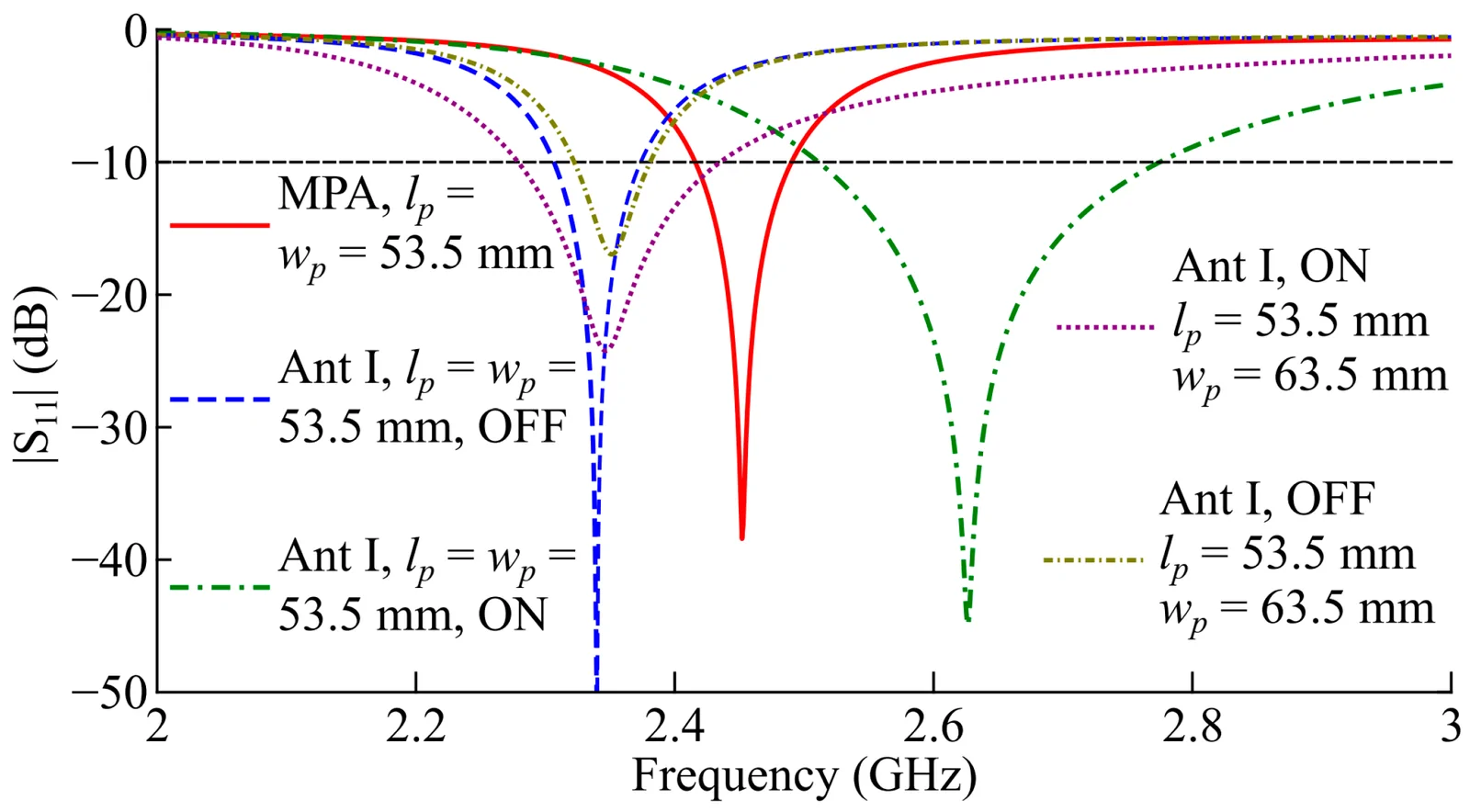
Simulated |S_11_| curves of square MPA and Ant I.

**Figure 3:**
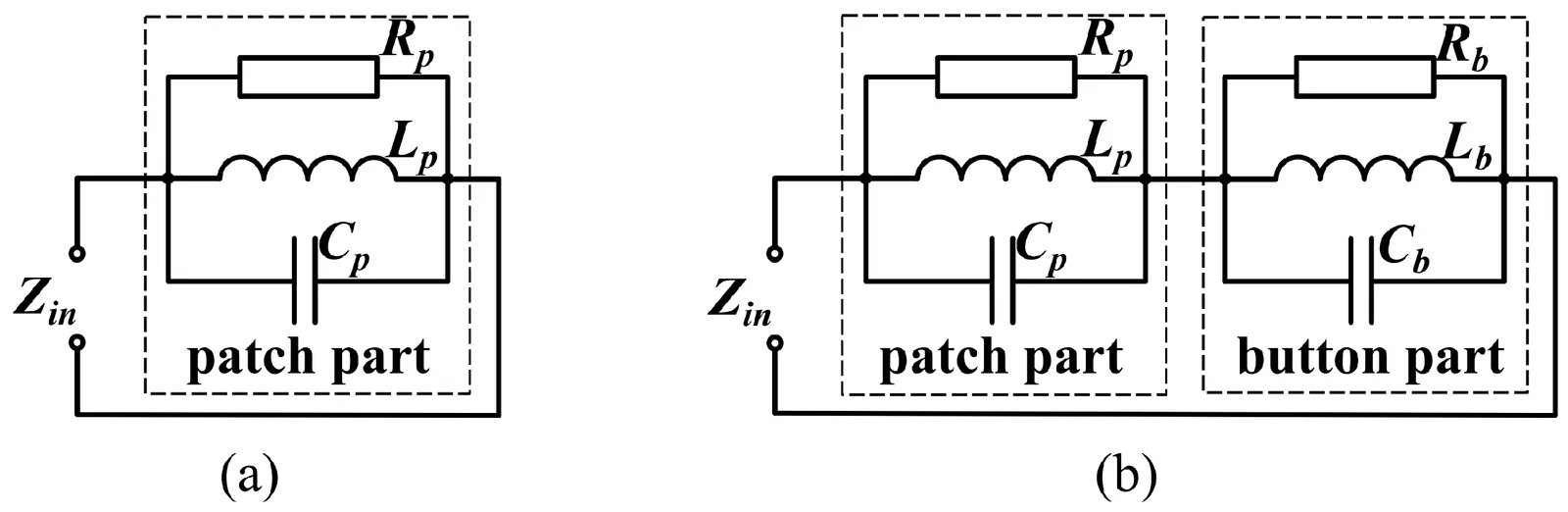
Equivalent circuits of Ant I with buttons (**a**) OFF and (**b**) ON.

**Figure 4:**
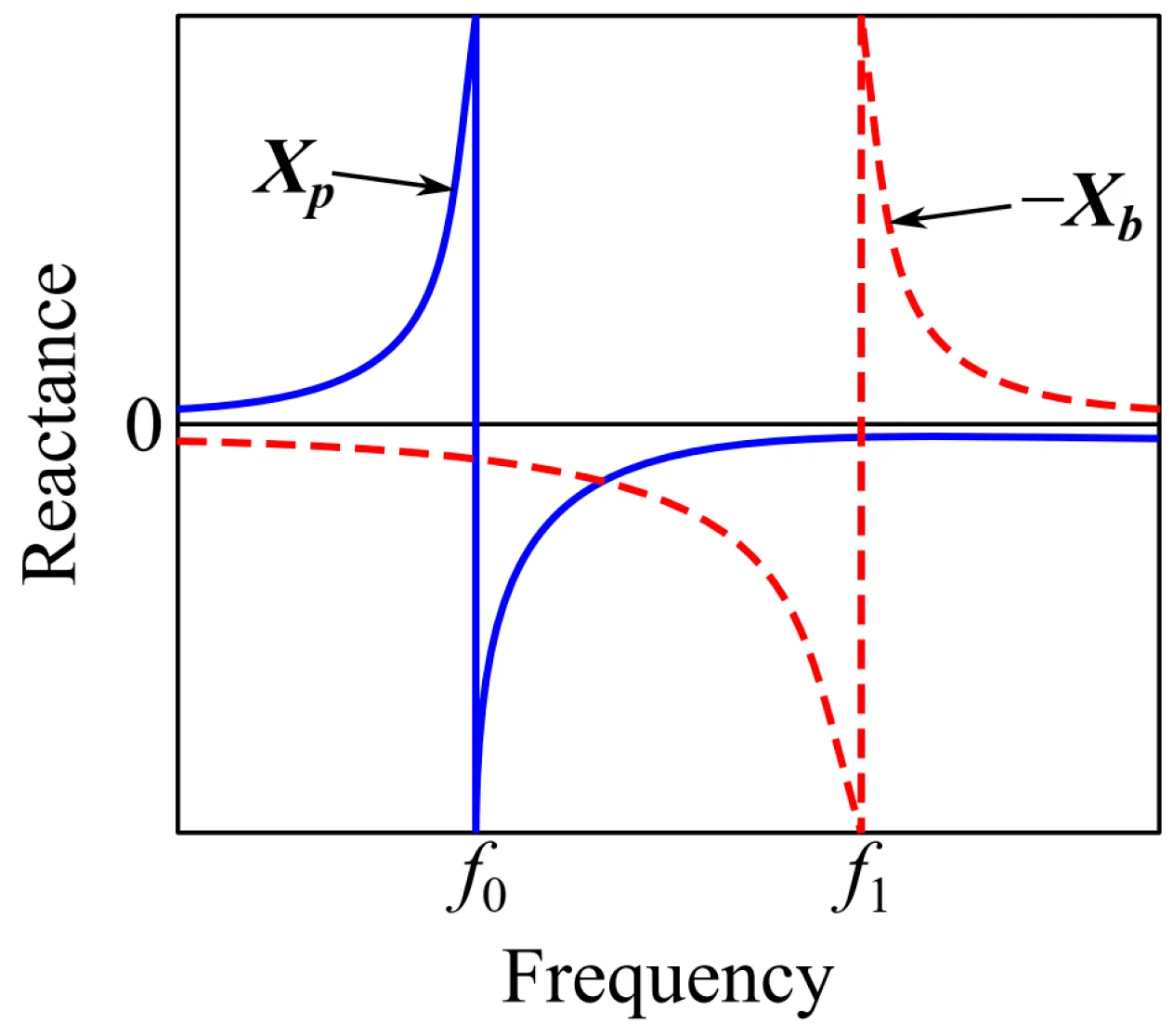
Graphical sketch of $X_{p}$ and −$X_{b}$ versus frequency.

**Figure 5:**
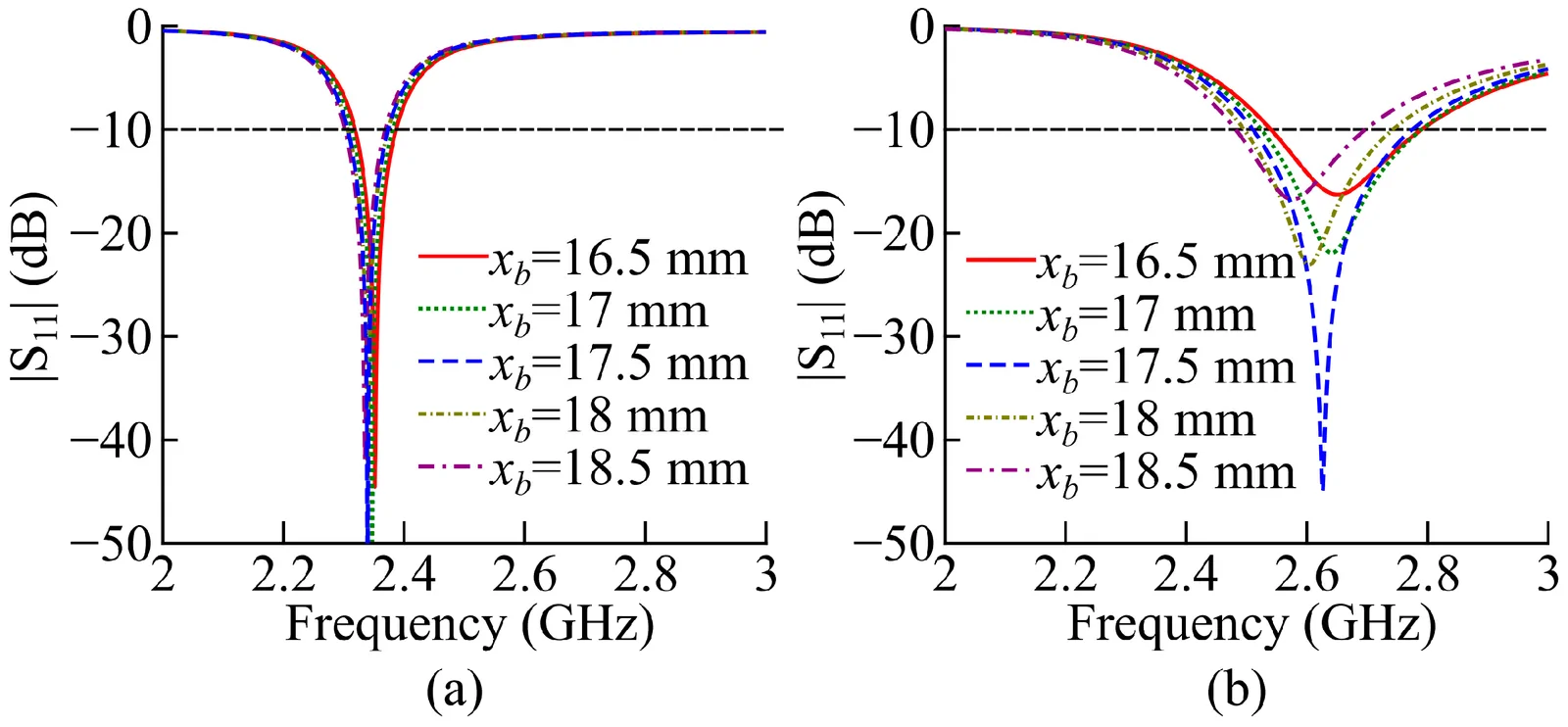
Simulated |S_11_| curves of Ant I with different *x_b_*: (**a**) buttons OFF; (**b**) buttons ON.

**Figure 6:**
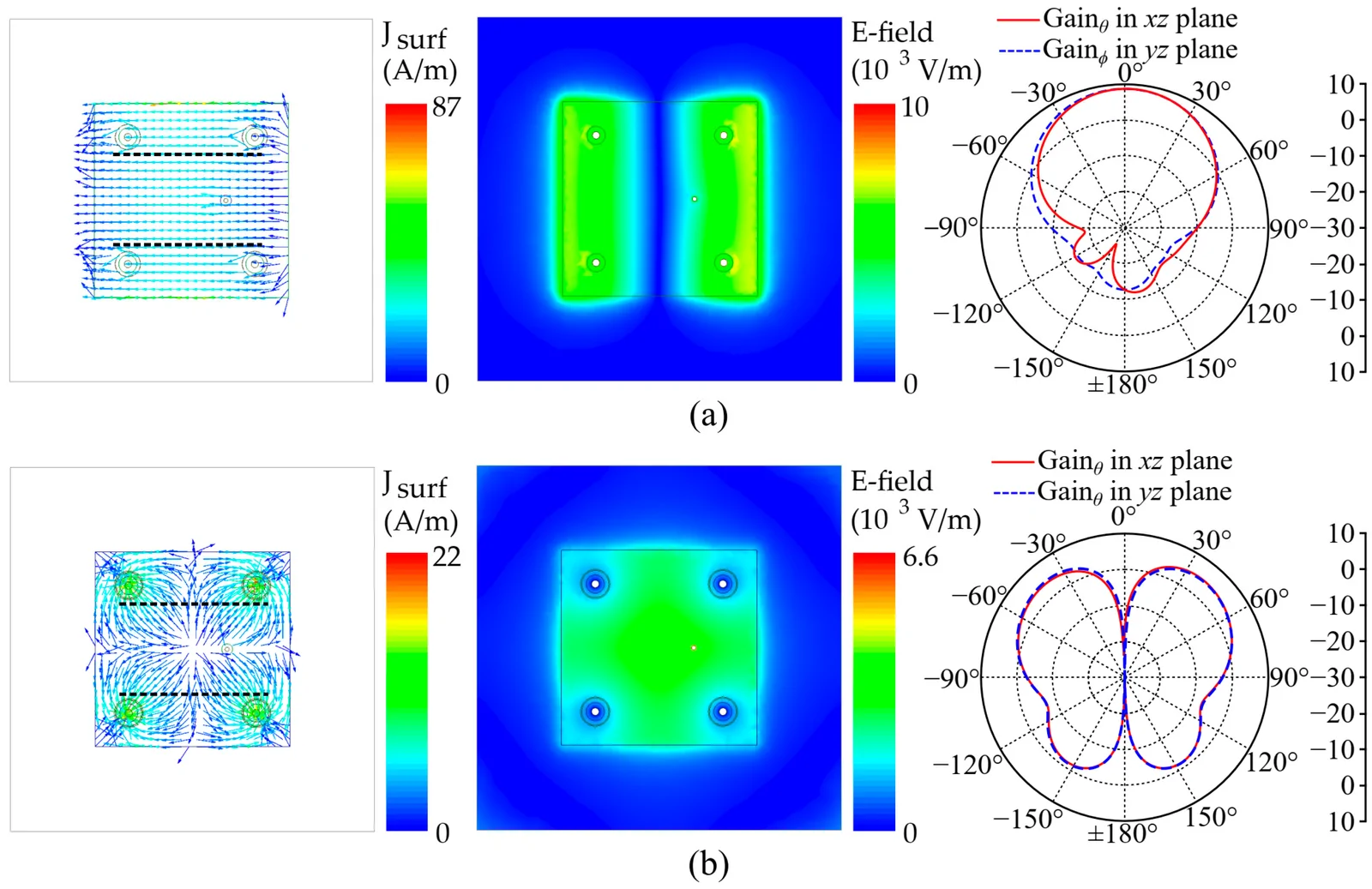
Simulated vector currents on the patch, internal E-field, and coplanar-polarized radiation patterns of Ant I: (**a**) at 2.34 GHz with buttons OFF; (**b**) at 2.63 GHz with buttons ON.

**Figure 7:**
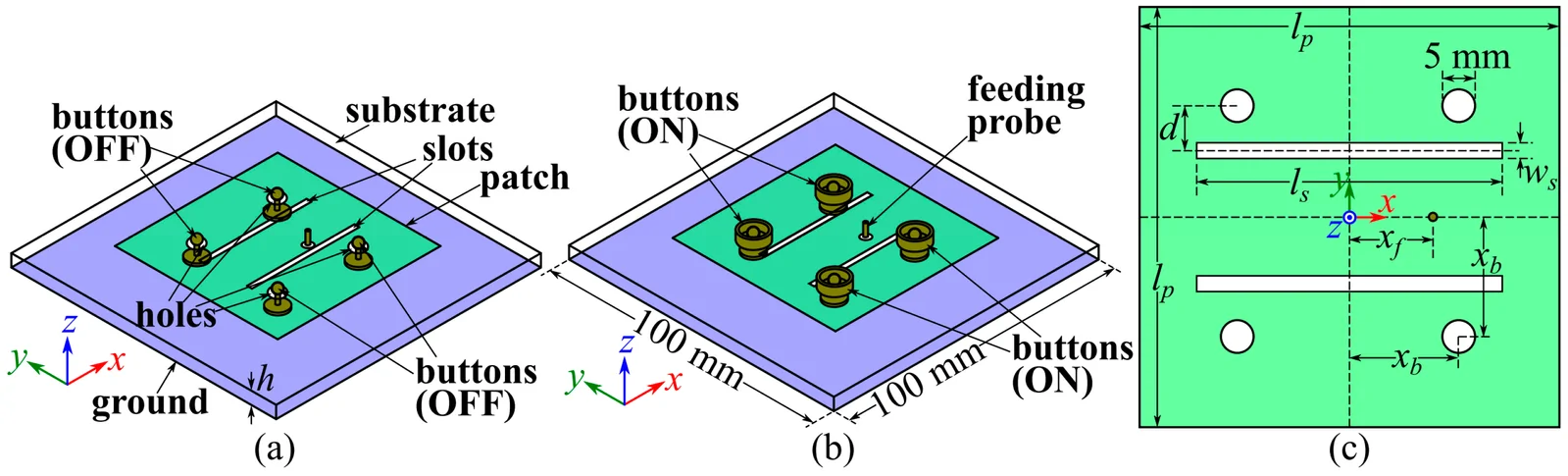
Geometry of Ant II: (**a**) buttons OFF; (**b**) buttons ON; (**c**) top view of patch.

**Figure 8:**
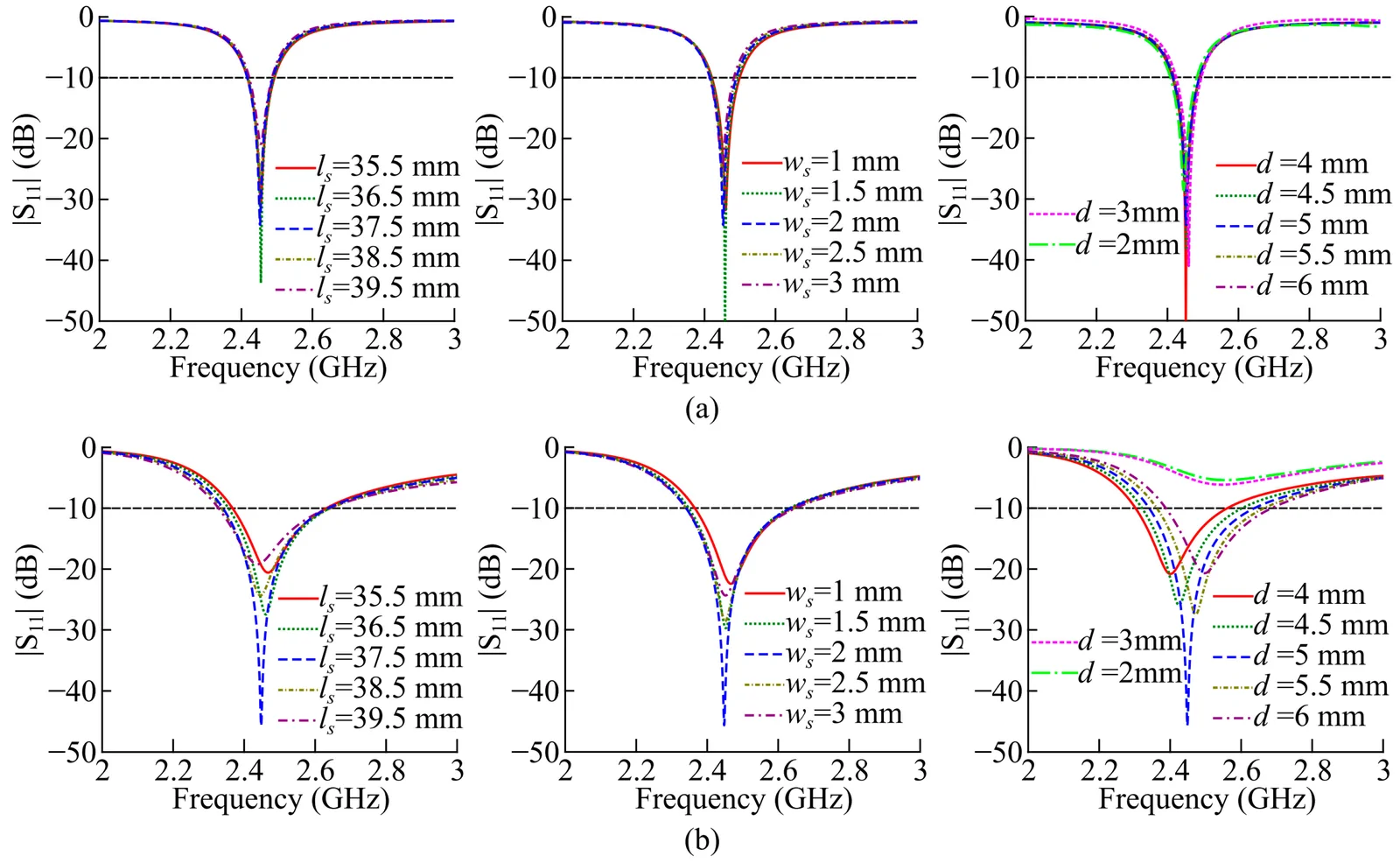
Simulated |S_11_| curves of Ant II with different *l_s_*, *w_s_*, and *d*: (**a**) buttons OFF; (**b**) buttons ON.

**Figure 9:**
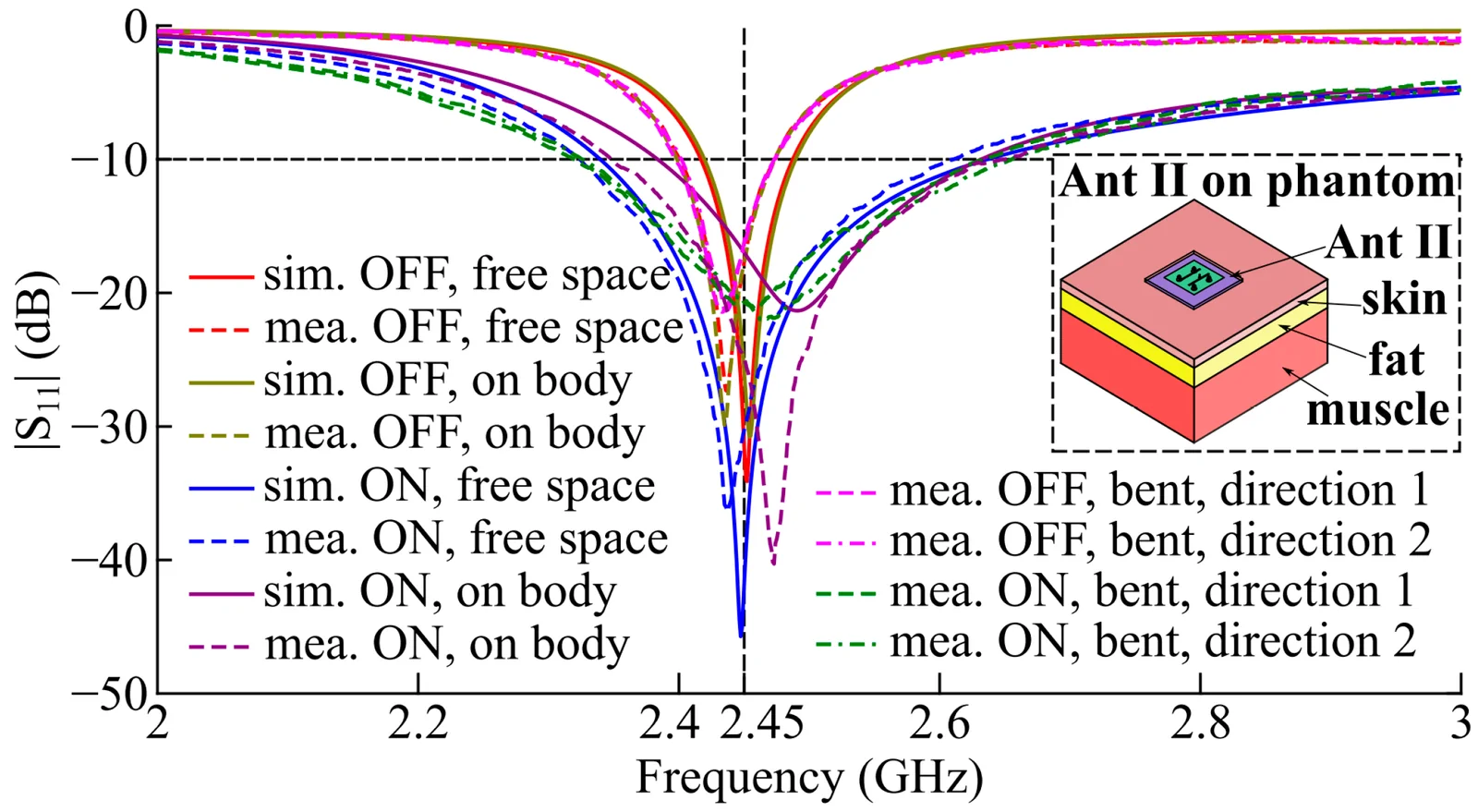
Simulated and measured |S_11_| curves of Ant II.

**Figure 10:**
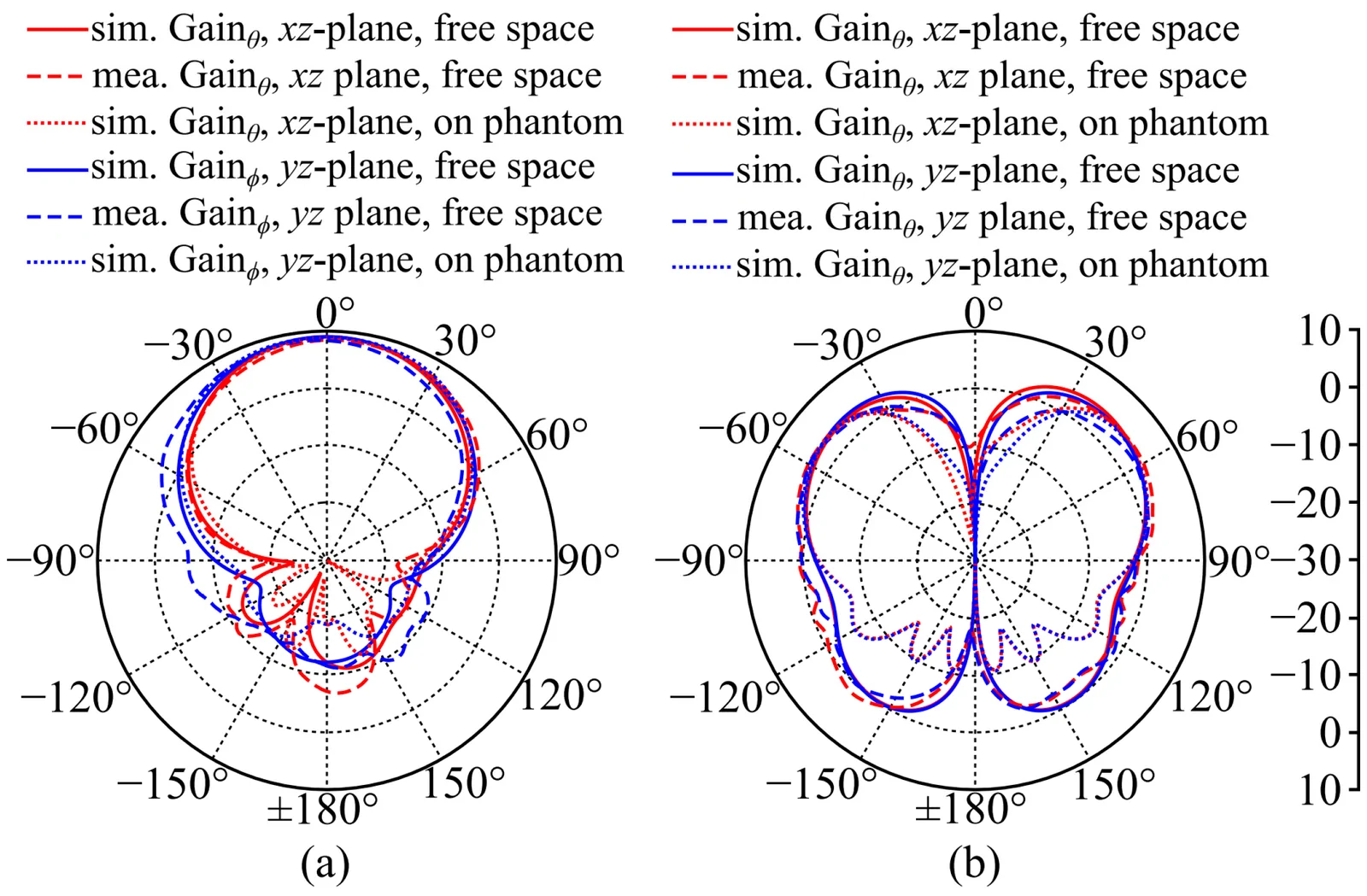
Simulated (at 2.45 GHz) and measured (at 2.44 GHz) coplanar-polarized gain patterns of Ant II: (**a**) buttons OFF; (**b**) buttons ON.

**Figure 11:**
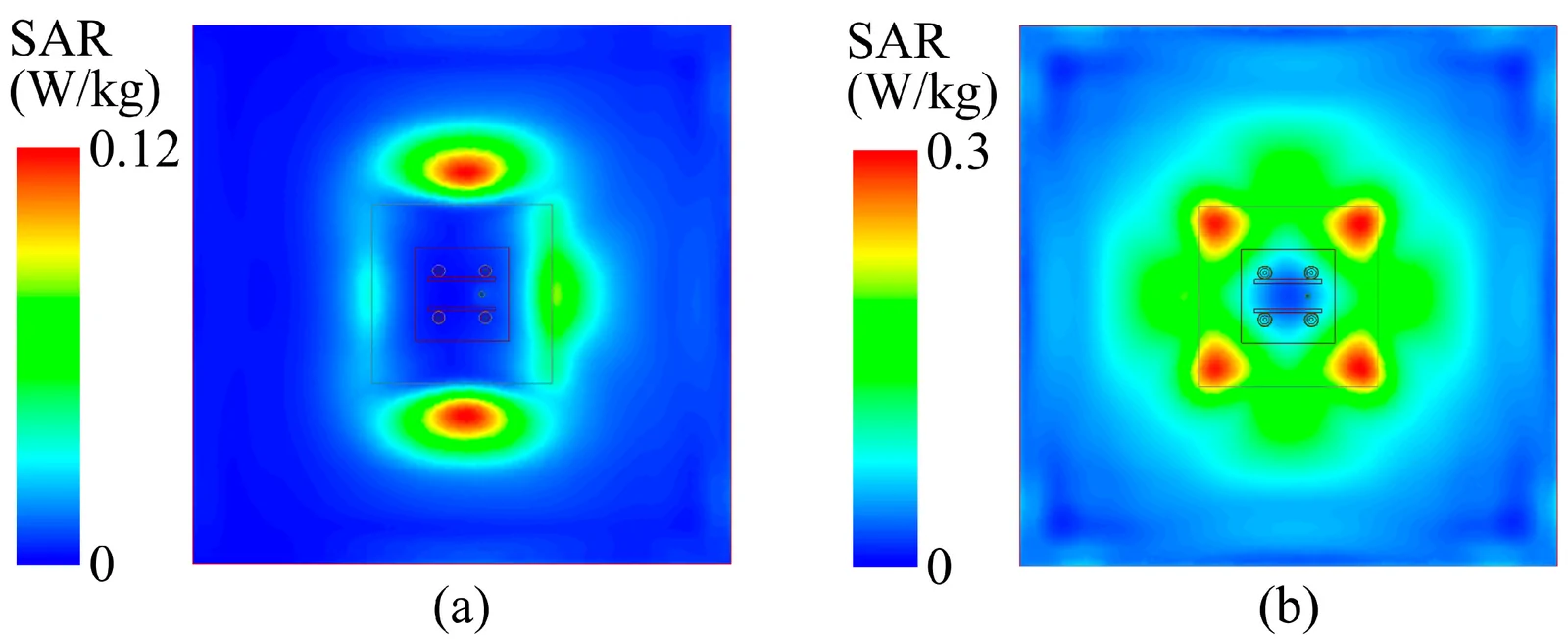
Simulated SAR distributions of Ant II at 2.45 GHz (0.5 W input power): (**a**) buttons OFF; (**b**) buttons ON.

**Figure 12:**
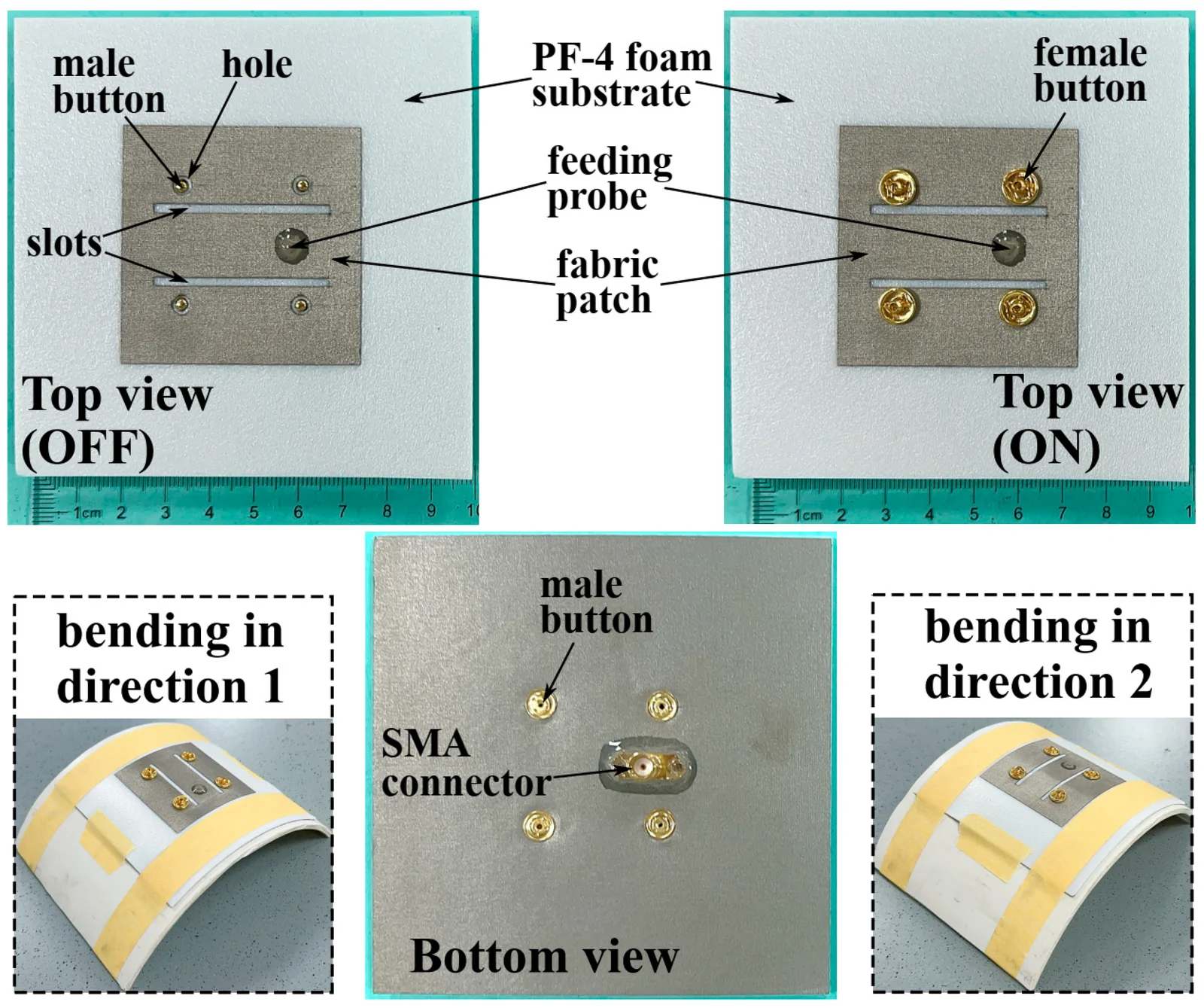
Photos of the fabricated prototype of Ant II.

**Figure 13:**
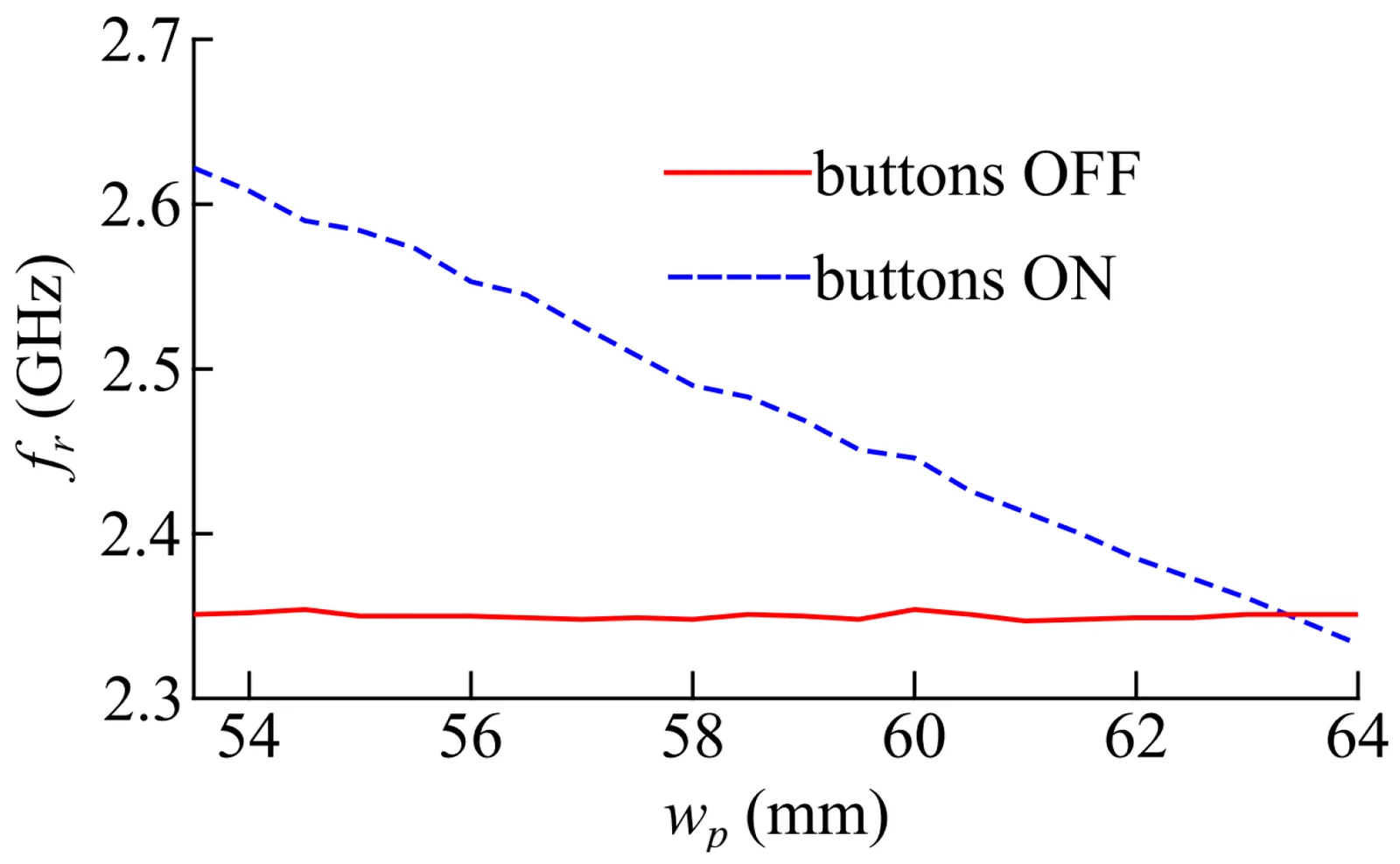
Simulated resonance frequencies of Ant I with respect to *w_p_*.

**Figure 14:**
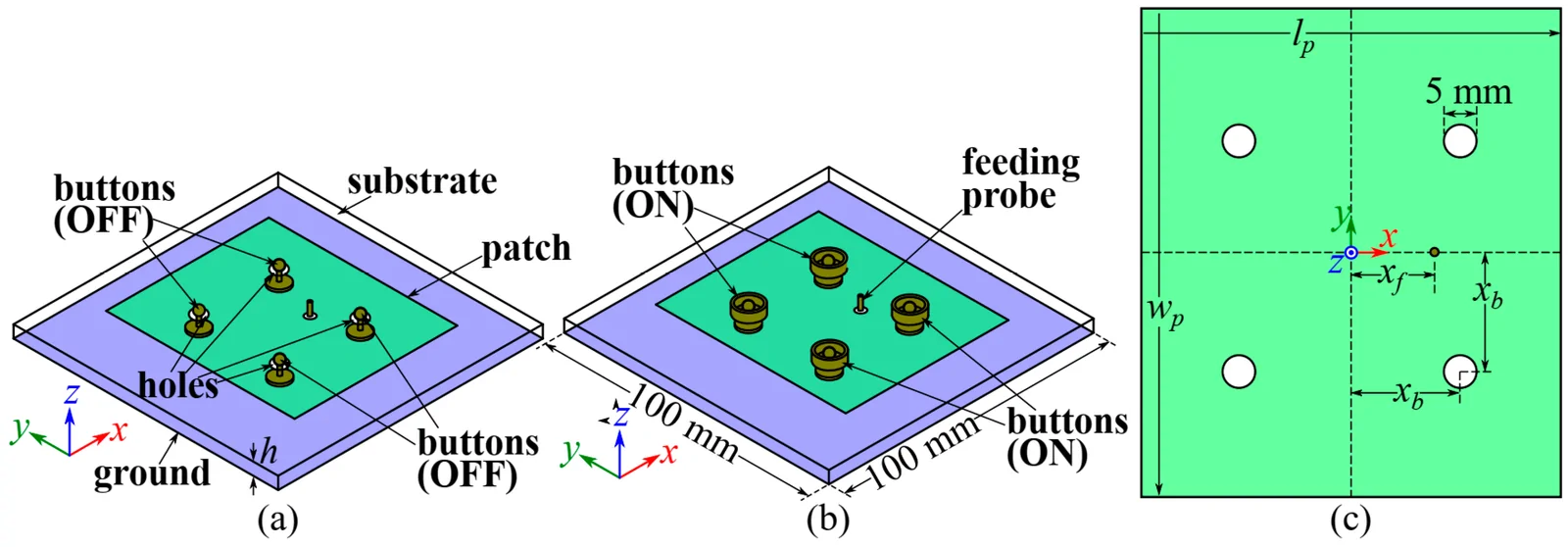
Geometry of Ant III: (**a**) antenna with buttons OFF; (**b**) antenna with buttons ON; (**c**) top view of patch.

**Figure 15:**
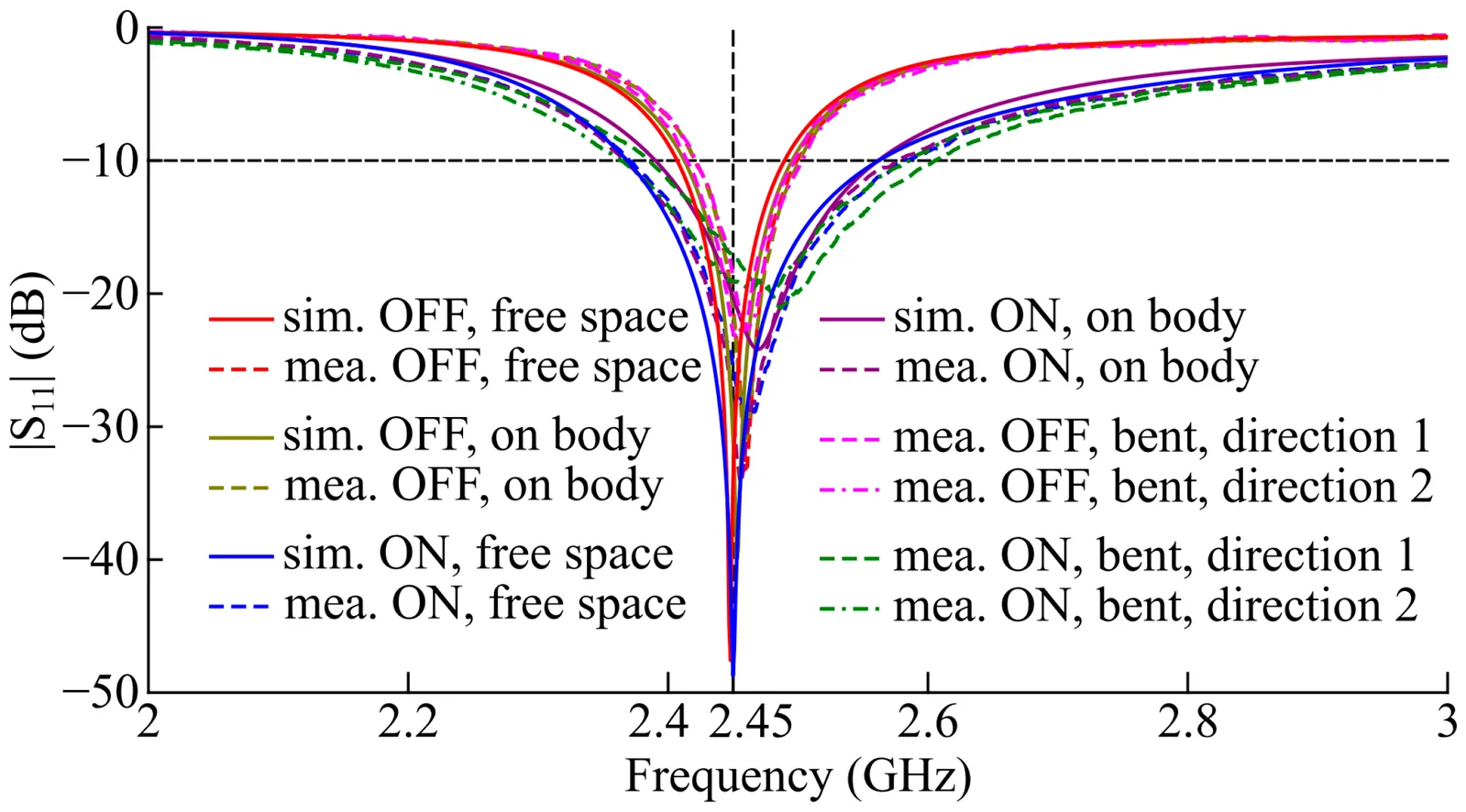
Simulated and measured |S_11_| curves of Ant III.

**Figure 16:**
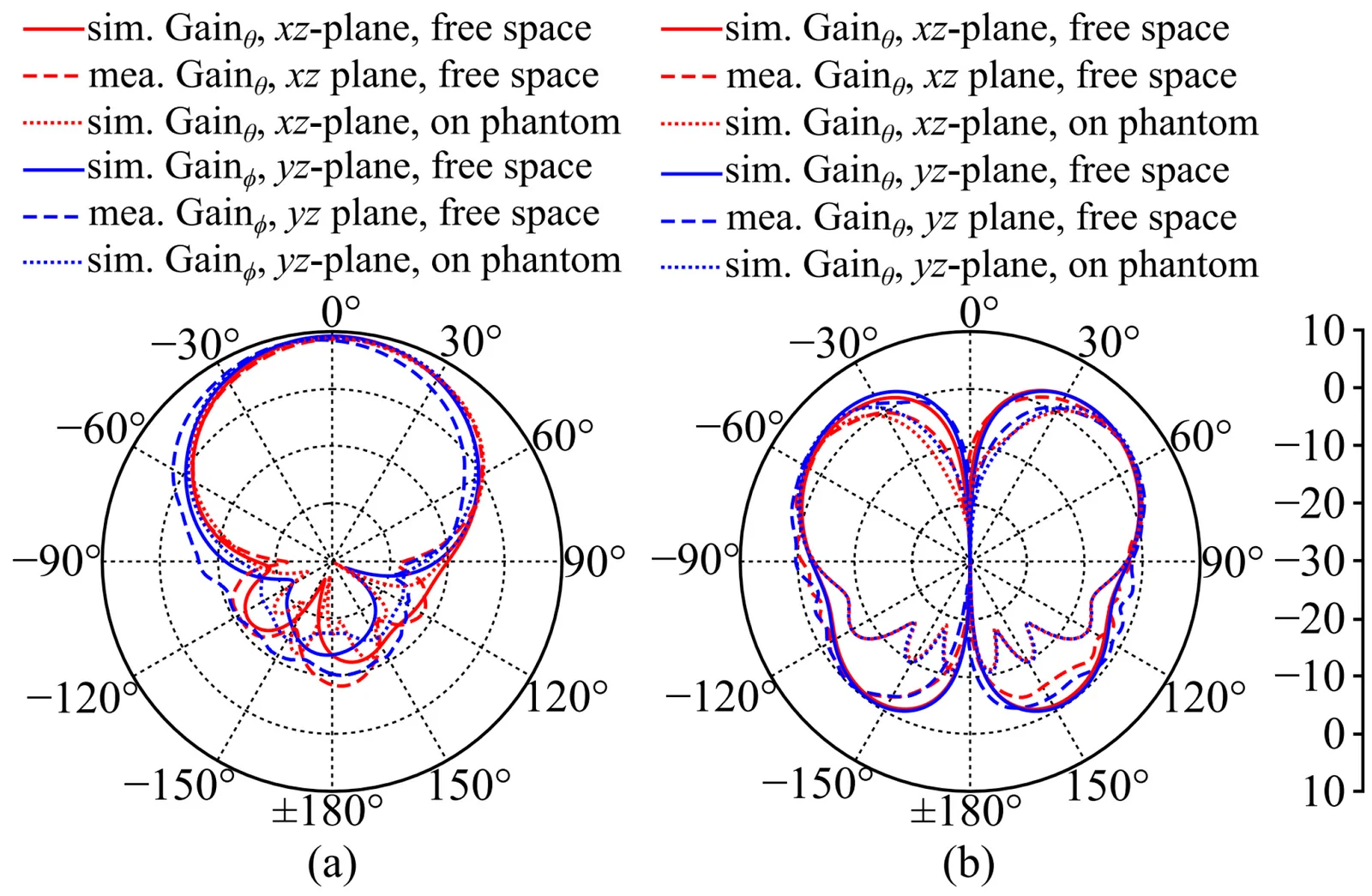
Simulated (at 2.45 GHz) and measured (at 2.46 GHz) coplanar-polarized gain patterns of Ant III: (**a**) buttons OFF; (**b**) buttons ON.

**Figure 17:**
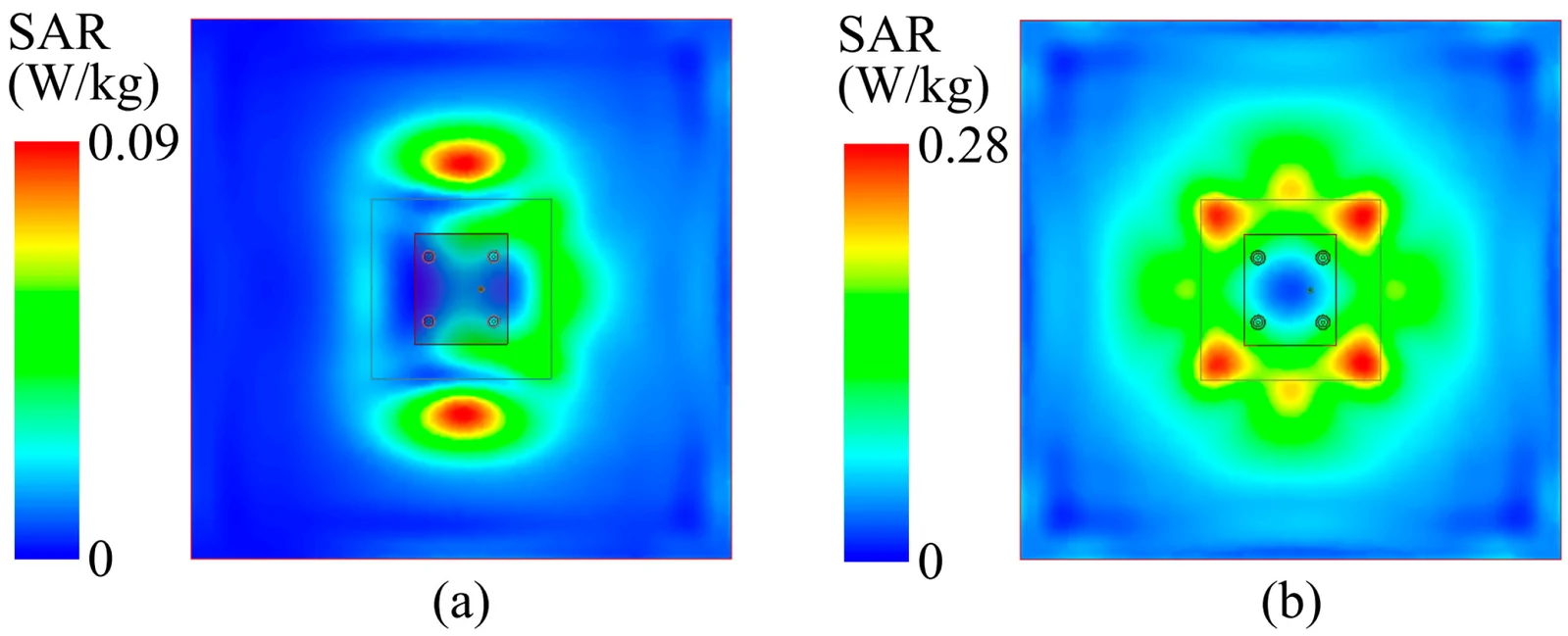
Simulated SAR distributions of Ant III at 2.45 GHz (0.5 W input power): (**a**) buttons OFF; (**b**) buttons ON.

**Figure 18:**
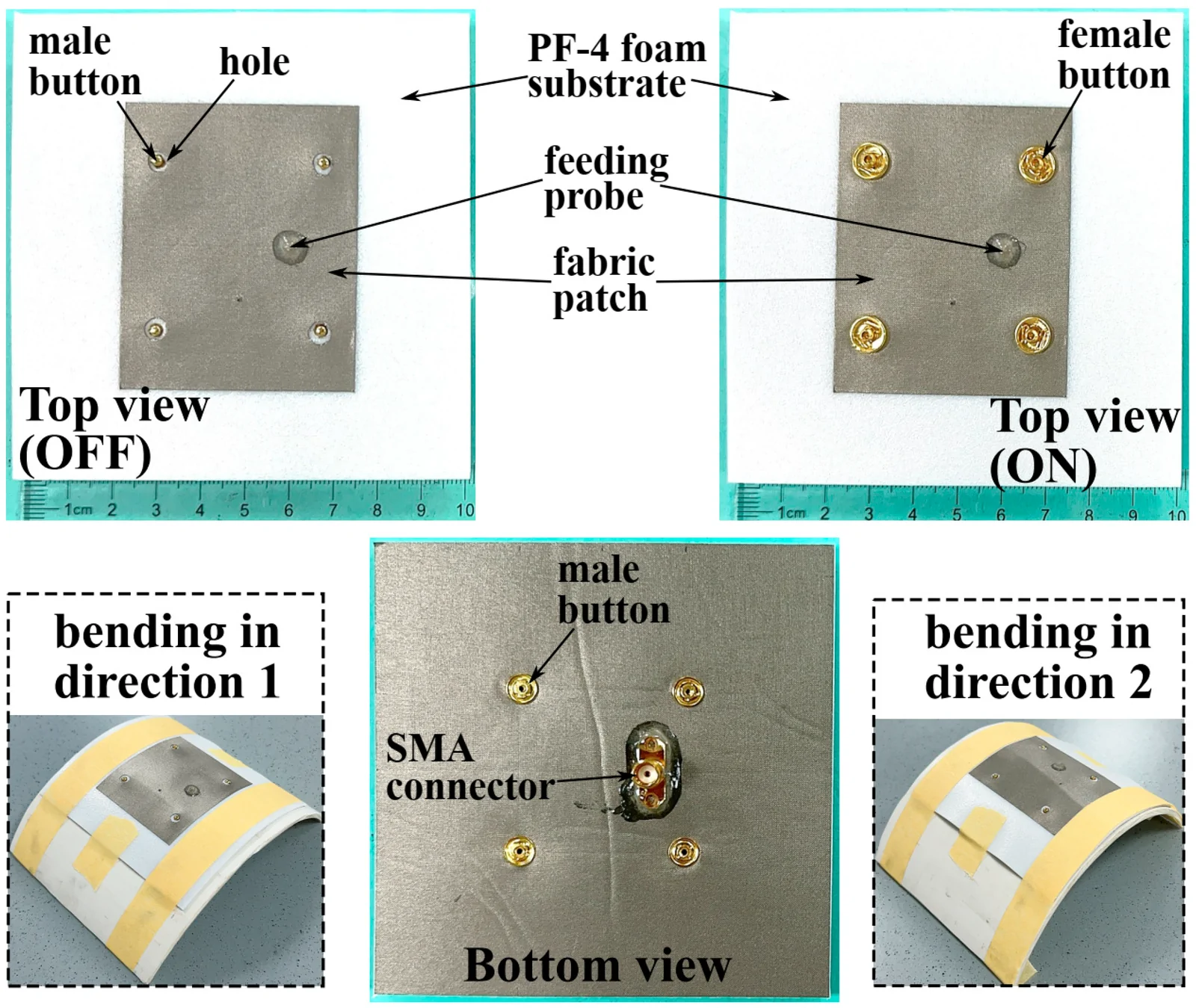
Photos of the fabricated prototype of Ant III.

**Table 1:** Dimensional parameters of proposed antennas.

Geometry	*l_p_* (mm)	*w_p_* (mm)	*x_f_* (mm)	*x_b_* (mm)	*l_s_* (mm)	*w_s_* (mm)	*d* (mm)
Ant I	53.5	53.5	9.5	17.5	-	-	-
Ant II	52	-	11	13	37.5	2	5
Ant III	51.1	61.5	11	15	-	-	-

**Table 2:** Thicknesses and characteristics of skin, fat, and muscle.

Layer	Thickness (mm)	Relative Permittivity	Conductivity (S/m)	Density (kg/m^3^)
Skin	3	38.09	1.43	1100
Fat	7	5.29	0.10	910
Muscle	50	52.82	1.69	1041

**Table 3:** Comparison with other wearable/rigid fixed-frequency pattern-adjustable patch antennas.

Reference	Patch Size ($\lambda_{\varepsilon }^{2}$)	Frequency (GHz)	Radiation Efficiency	Max Gain (dBi)
[16]	0.24	2.45	≥92% (sim.)	7.1/1.1 (sim.)
[17]	1.40	3.5	≥85% (mea.)	7.2/4.6 (mea.)
[18]	1.24	5.5	Not given	8.1/3.7 (mea.)
[19]	3.06	4.5	Not given	4.3/4.3 (mea.)
[20]	0.46	2.45	Not given	3.5/−2.6 (mea.)
	0.99	2.45	Not given	6.0/−2.5(mea.)
[21]	1.28	5.13	≥52% (mea.)	2.9/1.8 (mea.)
[22]	0.32	2.45	Not given	3.9/−4.3 (mea.)
[23]	0.24	2.45	≥76% (sim.)	4.9/−0.9 (mea.)
[24]	0.91	2.45	Not given	2.7/2.4 (mea.)
[25]	0.55	5.5	≥66% (mea.)	5.7/6.7 (mea.)
[26]	0.33	3.5	Not given	7.0/2.8 (mea.)
[27]	0.81	2.45	69% (mea.)	9.6/5.6 (mea.)
	0.79	2.45	64% (mea.)	8.7/5.3 (mea.)
[28]	0.45	2.45	≥91% (sim.)	9.2/3.1 (mea.)
Ant II	0.19	2.45	≥97% (sim.)	8.5/4.1 (mea.)
Ant III	0.22	2.45	≥99% (sim.)	8.8/3.8 (mea.)

## Data Availability

All data are included within the manuscript.
